# Endolysosomal two-pore channels regulate a conserved entry pathway for enteroviruses

**DOI:** 10.1099/jgv.0.002294

**Published:** 2026-07-15

**Authors:** Ta-Chou Weng, Bang-Yan Hsu, Tung-Heng Wu, Xin-Ya Gu, Hsu-Hung Cheng, Szu-Hao Kung

**Affiliations:** 1 Department of Biotechnology and Laboratory Science in Medicine, National Yang Ming Chiao Tung University, Taipei, Taiwan, ROC

**Keywords:** endolysosomal trafficking, enterovirus, two-pore channels, viral entry

## Abstract

Enteroviruses are significant human pathogens for which there are currently no effective antiviral therapies available. Two-pore channels (TPCs) are endolysosomal cation channels activated by nicotinic acid adenine dinucleotide phosphate (NAADP). These channels play a crucial role in regulating vesicular calcium (Ca²⁺) signalling and endolysosomal membrane trafficking. While endolysosomal TPCs have been implicated in the infectivity of some enveloped viruses, their role in non-enveloped virus infections remains unclear. In this study, we identify human TPC isoforms TPC1 and TPC2 as essential host factors that enhance enterovirus infection by regulating endolysosomal trafficking. Inhibiting NAADP-evoked Ca²⁺ signalling pharmacologically or using CRISPR/Cas9 to knockout either TPC isoform significantly suppressed the replication of enterovirus 71. Mechanistically, the loss of TPC function did not affect the viral binding, internalization or post-entry RNA translation and replication; instead, it delayed the viral progression through early and late endosomal compartments. Live-cell Ca²⁺ imaging confirmed that TPC-dependent NAADP signalling is necessary for endolysosomal Ca²⁺ release during infection. Our data also suggest cepharanthine, a bisbenzylisoquinoline alkaloid, inhibits enterovirus infection partly by modulating TPC-mediated Ca^2+^ signalling. Notably, the pharmacological inhibition or depletion of TPCs broadly restricted the replication of various enterovirus serotypes. Transcriptomic profiling revealed that the disruption of TPC1 or TPC2 alters gene networks associated with endocytosis and vesicular trafficking, emphasizing the vital role of TPCs in maintaining endolysosomal homeostasis. In summary, these findings establish TPC-dependent Ca^2+^ signalling as a conserved host pathway that enteroviruses exploit and highlight endolysosomal TPCs as promising targets for the development of broad-spectrum antiviral strategies.

## Introduction

Enteroviruses, members of the Picornaviridae family, are non-enveloped viruses possessing a positive-sense ssRNA genome. The genus *Enterovirus* encompasses many clinically important human pathogens, including polioviruses, coxsackievirus A and B groups, echoviruses, enteroviruses and rhinoviruses. Although most enterovirus infections are self-limited or asymptomatic, they can also precipitate severe, sometimes fatal diseases such as myocarditis, pancreatitis, meningitis, encephalitis and acute flaccid paralysis [1,2]. Among these agents, enterovirus 71 (EV71) is a major aetiological agent of hand, foot and mouth disease in infants and young children; a subset of cases progresses to serious neurologic complications, with fatal outcomes reported most frequently in the Asia–Pacific region [3]. At present, licensed vaccines are available for poliovirus and EV71 [4,5]; however, the broad antigenic diversity within the genus makes it difficult for any single vaccine to provide coverage against all enteroviruses. Compounding this challenge, there are still no approved drugs targeting enterovirus infections, underscoring the pressing demand for broad-spectrum antivirals capable of mitigating the wide-ranging and potentially life-threatening consequences of enteroviral disease.

The life cycle of enteroviruses begins with their specific binding to one or more cell surface receptors, thereby initiating the process of receptor-mediated entry [6]. This essential step varies according to the serotype of the virus and the particular host cell involved. Following the binding process, clathrin-mediated endocytosis facilitates viral trafficking, ultimately resulting in the release of the viral genome into the cytoplasm. Upon entering the cytosol, the positive-stranded viral genome serves as mRNA and undergoes translation to produce a polyprotein [7]. This translation process employs an internal ribosome entry site located in the 5′ untranslated region of the viral genome, enabling efficient, cap-independent translation [8]. The ensuing polyprotein is subsequently cleaved by virus-encoded proteases, specifically 2A and 3C, generating essential structural proteins known as VP1-4, in addition to critical non-structural proteins including proteases and polymerases. The replication of the viral genome is facilitated by the enzyme RNA-dependent RNA polymerase (3D) [9]. The newly synthesized positive-sense progeny viral RNAs are packaged into capsids, resulting in the formation of new virions. These virions are then released from the host cells through either lytic or non-lytic mechanisms [9].

Two-pore channels (TPCs) are cation channels located in the endolysosomal system and consist of two isoforms, TPC1 and TPC2, in mammals [10,11]. TPCs are essential for evoking localized Ca^2+^ fluxes triggered by nicotinic acid adenine dinucleotide phosphate (NAADP), which in turn initiates the fusion of vesicles such as endosomes and lysosomes through interaction with the soluble N-ethylmaleimide-sensitive factor attachment protein receptor protein machinery [12]. By regulating these fusion events, TPCs are thought to contribute to the control of cargo movement within the cellular trafficking system, potentially influencing processes ranging from early endosomal recycling to later stages associated with autophagic degradation [13,14]. TPC1 is primarily found on early and recycling endosomes and is involved in regulating endosomal trafficking and the transition from early to late endosomes. In contrast, TPC2 is mostly located in late endosomes and lysosomes, where it facilitates terminal fusion steps and supports lysosomal function [15,17]. The importance of TPCs is evident in various diseases; disrupted TPC function has been linked to neurodegeneration, cancer as well as metabolic and cardiovascular diseases, and may impair pathogen clearance [18]. Additionally, TPC1 and TPC2 are essential for the infection of certain enveloped viruses, including the Ebola virus and coronaviruses. Pharmacological inhibition or depletion of either TPC significantly reduces virus endolysosomal motility [19,21]. However, the specific role of TPCs in the infectivity of non-enveloped viruses, such as enteroviruses, has yet to be explored.

In this study, we demonstrate that pharmacological inhibition or CRISPR knockout (KO) suppresses EV71 infection by delaying viral trafficking and altering transcriptomic profiles associated with endocytosis. This suggests that TPC-dependent Ca^2+^ signalling is essential for efficient replication and represents a promising broad-spectrum antiviral target.

## Methods

### Cells and viruses

HeLa cells (ATCC CCL-2) and Rhabdomyosarcoma (RD) cells (ATCC CCL-13) were maintained in minimum essential medium (MEM) containing 10% FBS at 37 °C in a 5% CO_2_ incubator. Cells were seeded in 6-, 24- or 96-well plates to reach ~70–80% confluence on the day of the experiment. Enterovirus stocks used in this research included EV71 (BrCr strain), enterovirus 68 (EV68), coxsackievirus A16 (CVA16), CVB1, CVB2, CVB3 and Echovirus serotype 30 (Echo30) as documented [22].

### Generation of TPC1- and TPC2-knockout cell lines

KO of TPC1 or TPC2 was generated in the HeLa cell line using CRISPR/Cas9 genome editing following established methodologies [23]. The resulting cell lines were designated TPC1-KO and TPC2-KO, respectively. The pSpCas9(BB)−2A-Puro plasmid, a kind gift from Feng Zhang (Addgene plasmid #62988), was used to clone and express the guide RNA (gRNA) sequences targeting *tpc1* and *tpc2* genes, which are 5′-GATGTCCATGAAGGGCGGCAGGG-3′ [12] and 5ʹ-CCCCAGCGTCGGGCTGCTGG-3ʹ [24], respectively. HeLa cells were used to transfect with the gRNA-expressing plasmids. Selection of successfully transfected cells was achieved by the addition of puromycin to culture media. Single cells were isolated to ensure a clonal origin of the TPC1-KO and TPC2-KO cell lines. The KO levels of TPC1 and TPC2 were confirmed on protein levels via Western blot analysis.

### Chemicals

Trans-Ned-19 (CAS: 1354235-96-3), verapamil (CAS: 152-11-4), suramin (CAS: 129-46-4), tetrandrine (TET; CAS: 518-34-3) and cepharanthine (CEP; CAS: 481-49-2) were obtained from Cayman Chemical (Ann Arbor, MI, USA). All stock solutions of the compounds were prepared in DMSO and added to the cell culture medium. The final concentration of DMSO never exceeded 0.05%, which is well tolerated by all cell lines used in this study. Control samples without the drug were supplemented with an equivalent amount of 0.05% DMSO.

### Western blot

Cells were lysed in Radio-Immunoprecipitation Assay buffer (50 mM Tris/HCl pH 7.5, 150 mM NaCl, 1% NP-40, 0.5% sodium deoxycholate and 0.1% SDS) containing a protease inhibitor cocktail (Roche). Equal amounts of total protein (10–30 µg) were separated via SDS-PAGE and transferred to PVDF membranes (Millipore). Membranes were blocked in 5% nonfat dry milk in TBST (TBS +0.1% Tween-20) for 1 h at room temperature, then incubated overnight at 4 °C with primary antibodies that included mouse anti-EV71 VP1 (1 : 3,000, GTX633390, GeneTex), rabbit anti-α-tubulin (1 : 10,000, GTX112141, GeneTex), rabbit anti-TPC1 (1 : 3,000, TPCN1 Polyclonal Antibody PA5-90758, Invitrogen) and rabbit anti-TPC2 (1 : 500, ab119915, Abcam). After washing, membranes were incubated with HRP-conjugated goat anti-mouse or goat anti-rabbit secondary antibodies (1 : 3,000, Santa Cruz Biotechnology) for 1 h at room temperature. Signals were detected using an enhanced chemiluminescence reagent (GeneTex), and band intensities were quantified with ImageJ software.

### Virus infection and titration

Virus infections were performed in MEM supplemented with 2% FBS at 37 °C unless otherwise indicated. For virus titration, culture supernatants containing extracellular virus were collected and centrifuged at 5,700 × ***g*** for 5 min to remove cell debris. Cell-associated virus was prepared from infected cell lysates after three freeze–thaw cycles, followed by centrifugation at 15,300 × ***g*** for 10 min. Total virus represents the combined mixture of extracellular and cell-associated virus fractions. Infectious titres were determined on RD cells by the 50% TCID_50_ method using the Reed–Muench calculation [25].

### Intracellular Ca^2+^ measurements

HeLa, TPC1-KO and TPC2-KO cells were pretreated for 1 h at 37 °C with *trans*-Ned-19 (100 µM), verapamil (100 µM), TET (20 µM), CEP (20 µM) or with vehicle control (0.05% DMSO). Cells were then incubated with Fluo-8 AM (5 µM; AAT Bioquest) for 30 min at 37 °C in Hanks’ balanced salt solution containing 1 mM CaCl_2_. After washing, cells were stimulated with 1 µM of NAADP-AM (Cellular and Molecular Technologies), and real-time fluorescence was recorded every 10 s for up to 3 min using a fluorescence microscope (Leica DM6000B; excitation ~490 nm, emission ~525 nm). Fluorescence intensity was quantified using ImageJ software. Data were normalized to baseline fluorescence (F₀) and expressed as F/F₀ over time.

### RNA isolation and quantitative reverse transcription-PCR

Total cellular and viral RNA was prepared essentially as described previously [26]. Briefly, cells were lysed and RNA was isolated using TRIzol reagent (Thermo Fisher Scientific) according to the manufacturer’s protocol. cDNA was generated with AMV Reverse Transcriptase XL (Takara), and quantitative PCR was carried out using the FastStart Universal SYBR Green Master mix (Roche Applied Science) on a real-time PCR platform. Primer pairs specific for the VP1 region of the EV71 (BrCr strain) genome and for human β-actin have been reported previously.

### Virus binding and internalization assays

Virus binding and internalization assays were conducted as previously reported [27]. HeLa, TPC1-KO and TPC2-KO cells were seeded in six-well plates 1 day prior to infection. Cells were pretreated with suramin (40 or 80 µM) or DMSO for 2 h at 37 °C. After pretreatment, cells were incubated for 10 min in 1 ml binding buffer (PBS containing 1% BSA and 0.1% sodium azide) at 4 °C. EV71 was then added at an m.o.i. of 50 and allowed to adsorb for 1 h at 4 °C. For the binding assay, unbound virus was removed, and the cells were washed extensively with ice-cold PBS before being collected by scraping them for RNA extraction and reverse transcription-quantitative PCR (RT-qPCR) quantification of EV71 RNA. In the internalization assay, after the 4 °C adsorption step, unbound virus was removed and replaced with pre-warmed complete medium. The cells were then shifted to 37 °C for 1 h to allow for internalization. Following this, the cells were briefly treated with trypsin to remove any residual surface-bound virions, neutralized with complete medium, washed extensively with ice-cold PBS and harvested for RNA extraction and RT-qPCR quantification

### EV71 movement analysis

HeLa, TPC1-KO and TPC2-KO cells were seeded on glass coverslips in 24-well plates and infected with EV71 at an m.o.i. of 300 at 4 °C for 1 h to allow synchronized virus binding. After adsorption, the unbound virus was removed by three washes with PBS, and the cells were shifted to 37 °C in complete medium for 15, 30, 45, 60 or 90 min. At each time point, cells were fixed with 4% paraformaldehyde and permeabilized with 0.2% Triton X-100. Viral particles were visualized using a rabbit polyclonal antibody against EV71 VP2 (1 : 2,000; GTX132340, GeneTex) followed by a goat anti-rabbit IgG (H+L) secondary antibody conjugated to Alexa Fluor 488 (1 : 500; ab150077, Abcam). Cells were co-stained for endosomal markers using EEA1 (early endosome antigen 1) Alexa Fluor 546 antibody (G-4, 1 : 500; sc-137130, Santa Cruz) to identify early endosomes. Cells were co-stained with LAMP1 (lysosomal-associated membrane protein 1) Alexa Fluor 546 antibody (H4A3, 1 : 500; sc-20011, Santa Cruz) to label late endosomes and lysosomes. The nuclei were counterstained with DAPI. Images were acquired with a confocal laser scanning microscope (Carl Zeiss LSM 880 META). Green fluorescence indicated viral VP2, red fluorescence corresponded to EEA1 or LAMP1 and blue fluorescence marked nuclei. The percentage of colocalization between EV71 VP2 and EEA1 or LAMP1 was quantified using ZEN 2 software.

### EV71 replicon assay

The EV71 subgenomic replicon plasmid pSVA-EV71-GFP [28], encoding a GFP reporter, was linearized with *NotI* endonuclease and used as a template for *in vitro* transcription with the RiboMAX™ Large Scale RNA Production System-T7 (Promega, P1320), as described previously [27]. The transcribed RNA was precipitated with LiCl and washed with ice-cold 70% ethanol. For each well, 2,000 ng of EV71-GFP RNA and 500 ng of pDsRed2-N1 plasmid DNA (Clontech) were co-transfected using Lipofectamine™ 2000 (Thermo Fisher Scientific) according to the manufacturer’s protocol. After 5 min, the transfection reagent was removed and replaced with fresh medium supplemented with 10% FBS. At 18 h post-transfection, cells were examined using a fluorescent microscope (Leica DMi6000B) equipped with FITC and TRITC filter sets to detect GFP and DsRed2 signals, respectively. Image analysis was performed using ImageJ software.

### RNA sequencing

Total RNA was isolated from HeLa, TPC1-KO and TPC2-KO cells using the RNeasy Mini Kit (Qiagen, Hamburg, Germany) according to the manufacturer’s instructions. The integrity and quantity of the RNA were assessed using microfluidic electrophoresis and spectrophotometry to ensure high-quality RNA profiles for RNA sequencing (RNA-Seq). The mRNA-Seq libraries were prepared using the Universal Plus mRNA-Seq kit (Tecan) according to the manufacturer’s protocol. Paired-end sequencing (2×150 bp) was conducted on a NovaSeq 6000 platform (Illumina, San Diego, CA). The sequencing reads were aligned to the GRCh38 human reference genome using CLC Genomics Workbench v10 (Qiagen).

### Differential expression and pathway analysis

For each gene that was annotated, we obtained read counts and normalized the expression levels as fragments per kilobase of transcript per million mapped reads. Genes were classified as differentially expressed if they exhibited a minimum fold change of 2 between KO and parental cells. To perform gene ontology (GO) and pathway enrichment analyses, we used the Database for Annotation, Visualization and Integrated Discovery (DAVID) available at https://davidbioinformatics.nih.gov/ [29,30]. Biological processes and KEGG Kyoto Encyclopedia of Genes and Genomes pathways were considered significantly enriched if their *P*-values were ≤0.05.

## Results

### Inhibitors of TPC-dependent NAADP signal effectively suppress EV71 infection

**Fig. 1.**
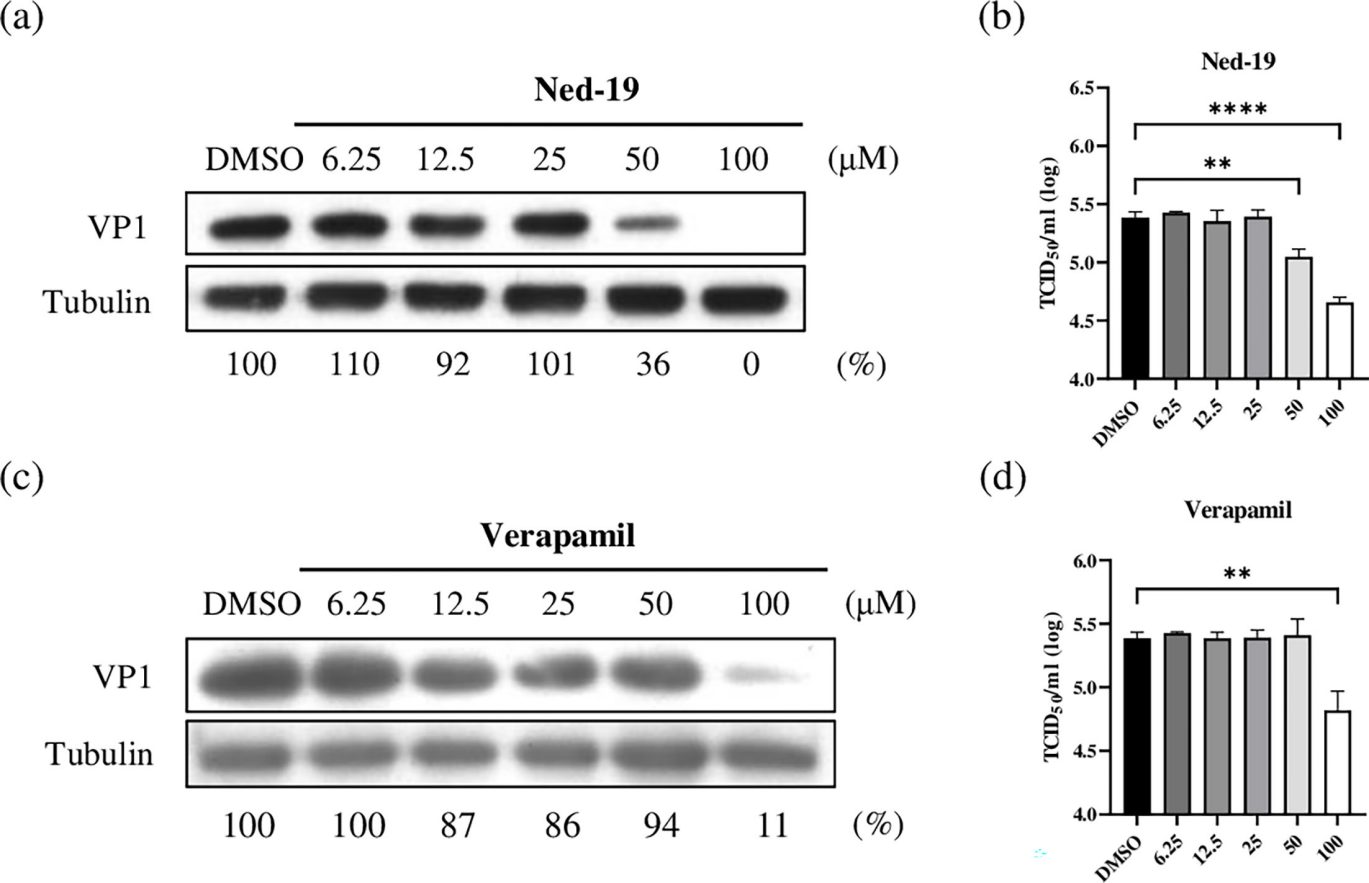
Pharmacological inhibition of TPC-dependent Ca^2+^ signalling reduces EV71 infection. HeLa cells were pretreated with Ned-19 (**a, b**) or verapamil (**c, d**) at the indicated concentrations for 1 h, followed by infection with EV71 at an m.o.i. of 0.5, with the corresponding compounds present for an additional 6 h. (**a, c**) Cell lysates were prepared and subjected to Western blot analysis using an anti-EV71 VP1 antibody and an anti-tubulin antibody as an internal control. The percentages below each lane represent VP1 band intensities normalized to tubulin levels. (**b, d**) At 8 h post-infection, cell lysates and supernatants were collected, and total virus titres were determined by the TCID_50_ assay. Data are presented as mean±sd of three independent experiments. Statistical significance was evaluated by one-way ANOVA; ****P<0.0001, **P<0.01.

Previous studies have demonstrated that TPC1- and TPC2-dependent NAADP signalling is essential for the entry of certain enveloped viruses, i.e. Ebola virus and coronaviruses [19,20]. To investigate whether either TPC is also involved in the replication of the non-enveloped enteroviruses, we first examined the effects of small-molecule inhibitors that target NAADP-evoked Ca^2+^ signalling during EV71 infection. HeLa cells were treated with Ned-19, a widely used chemical antagonist of NAADP signalling [31], or verapamil, a clinically used l-type Ca^2+^ channel blocker known to interfere with NAADP-evoked Ca^2+^ release [32], both at non-cytotoxic concentrations (Fig. S1a, b, available in the online Supplementary Material). Western blot analysis revealed that Ned-19, at concentrations of 50 and 100 µM, reduced viral VP1 levels to less than 40% of the control and even rendered them undetectable, respectively (Fig. 1a). Additionally, verapamil at a concentration of 100 µM reduced VP1 levels to ~11% of the control (Fig. 1c). Consistent with the findings, viral titres were also significantly reduced by the treatment of Ned-19 (Fig. 1b) and verapamil (Fig. 1d). These data collectively indicate that NAADP-evoked TPC-dependent Ca^2+^ signalling is required for efficient EV71 replication.

### Genetic ablation of TPC1 or TPC2 impairs EV71 infection

**Fig. 2.**
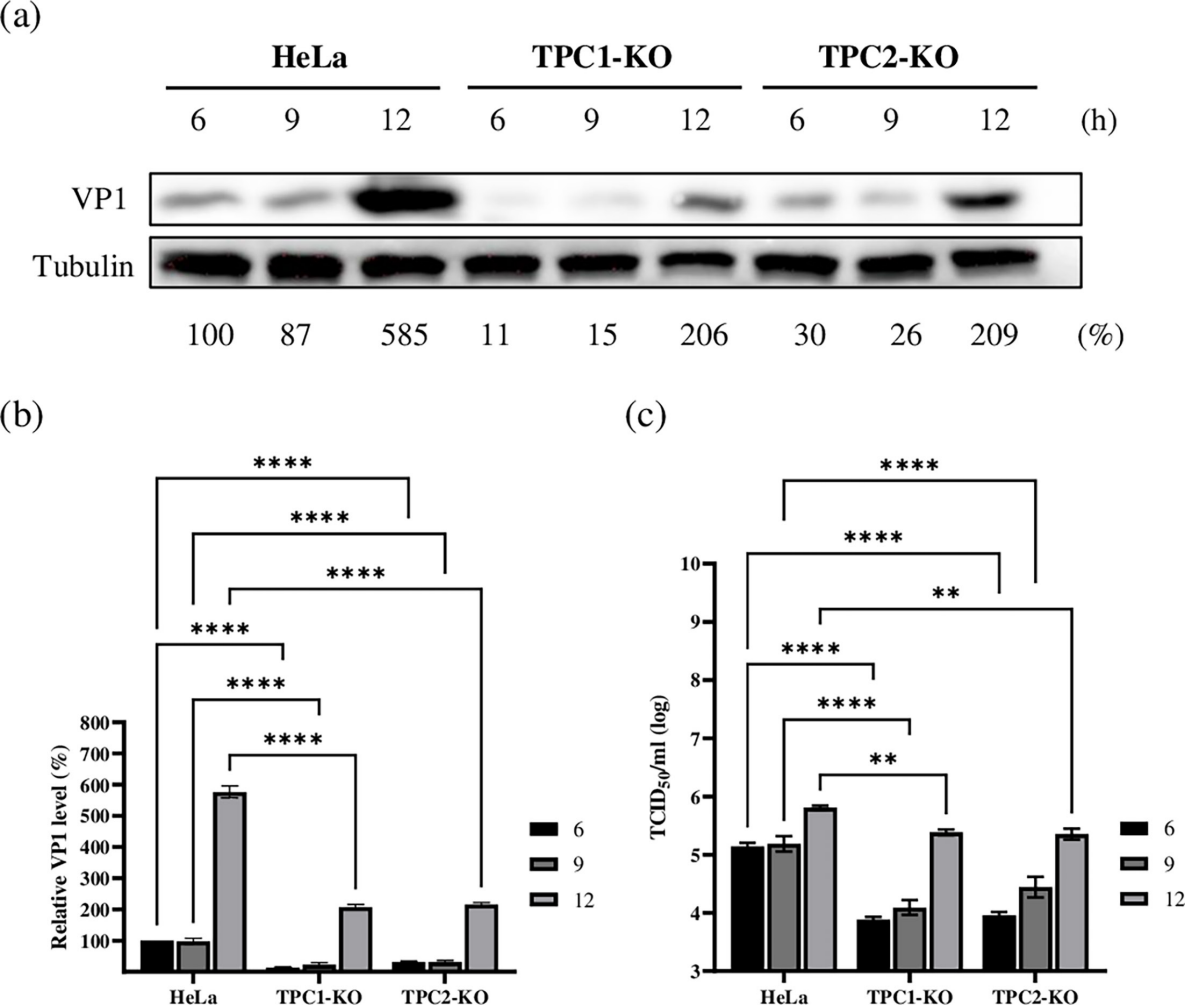
Genetic KO of TPC1 or TPC2 impairs EV71 replication. Parental HeLa, TPC1-KO and TPC2-KO cells were infected with EV71 at an m.o.i. of 0.5 and harvested at 6, 9 and 12 h post-infection. (**a**) Cell lysates were prepared, and Western blotting was used to detect the viral VP1 protein alongside tubulin, which served as a loading control. The numbers below the VP1 bands represent densitometric quantification normalized to tubulin, expressed as a percentage relative to the parental HeLa cells at 6 h post-infection (set to 100%). (**b**) The quantification of VP1 protein levels from three independent experiments is presented as relative VP1 levels (%) at the specified time points. (**c**) The viral titres were determined using the TCID_50_ assay. Data are presented as mean±sd (*n*=3). Statistical significance was evaluated by one-way ANOVA; ****P<0.0001, **P<0.01.

We next investigated whether either TPC isoform is required for EV71 infection by generating KO cells for TPC1 and TPC2, referred to as TPC1-KO and TPC2-KO, respectively, using CRISPR/Cas9-mediated genome editing. Both KO cell lines exhibited similar morphology and growth rates compared to their parental HeLa cells (Fig. S2a, b). Western blot analysis revealed that TPC1 and TPC2 proteins were nearly undetectable in the TPC1-KO and TPC2-KO lines, respectively. Interestingly, the protein levels of the non-targeted isoform were modestly higher than those in the parental cells, suggesting a possible compensatory upregulation (Fig. S2c). Subsequently, we infected TPC1-KO, TPC2-KO and their parental HeLa cells with EV71 for durations of 6, 9 and 12 h. Throughout the infection period, we observed substantial reductions in the levels of the viral VP1 protein (Fig. 2a, b) and viral titres (Fig. 2c) in the KO cells compared to their parental counterparts. These findings support our pharmacological observations and highlight the essential roles of both TPC1 and TPC2 in EV71 replication.

### NAADP-pathway inhibitors and depletion in either TPC attenuate NAADP-evoked Ca^2+^ signals

**Fig. 3.**
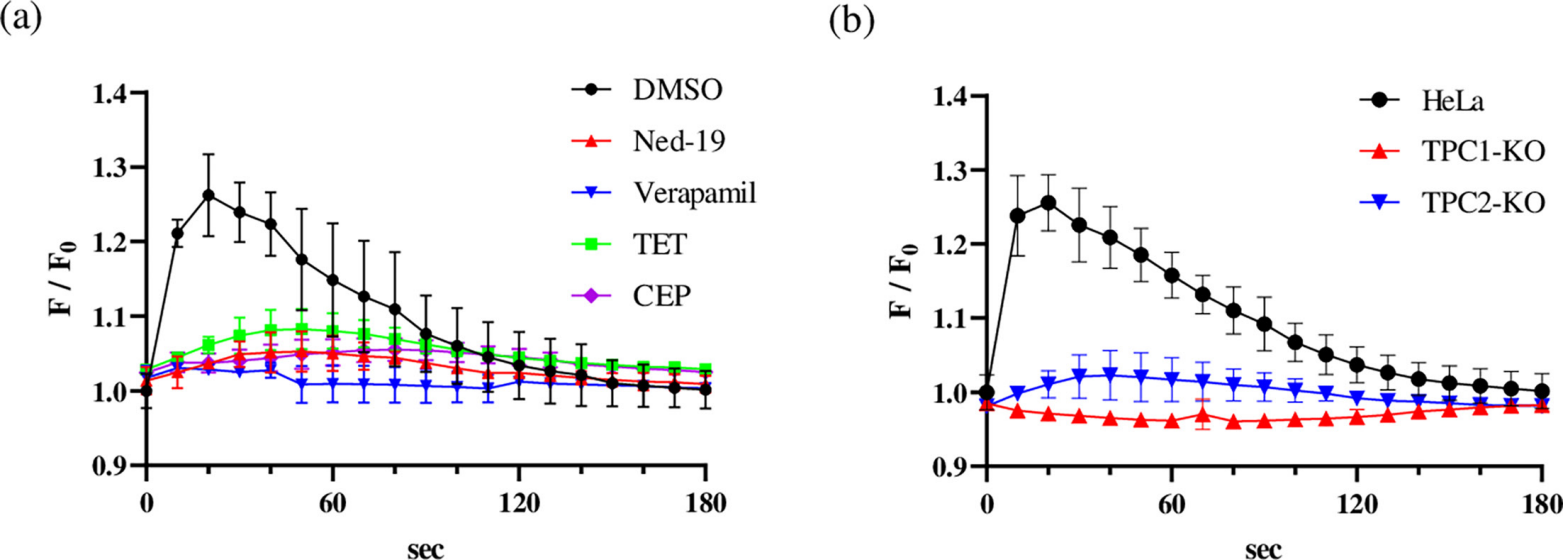
NAADP-AM-evoked Ca^2+^ signals are attenuated by pharmacological NAADP-pathway inhibitors and by genetic KO of either TPC. (**a**) Parental HeLa cells were pretreated for 1 h with Ned-19 (100 µM), verapamil (100 µM), TET (20 µM) or CEP (20 µM), then loaded with Fluo-8 AM for 30 min, and subsequently stimulated with 1 µM NAADP-AM. (**b**) Parental HeLa, TPC1-KO and TPC2-KO cells were loaded with Fluo-8 AM for 30 min and stimulated with 1 µM NAADP-AM. Fluorescence images were acquired every 10 s for 3 min. Mean fluorescence intensity (**f**) at each time point was quantified and normalized to the baseline fluorescence prior to stimulation (F₀) and plotted as F/F₀. Fluorescence intensity was quantified and analysed using ImageJ. Data are presented as mean±sd from independent experiments (*n*=3).

We have shown that pharmacological disruption of NAADP signalling, along with the genetic KO of TPC1 or TPC2, significantly reduces EV71 infection (Figs1  2). Next, we investigated whether these interventions also diminish NAADP-evoked Ca^2+^ release, a key indicator of TPC-dependent signalling from acidic organelles [33]. To explore this, we monitored cytosolic Ca^2+^ responses induced by NAADP-AM under various pharmacological treatments. NAADP-AM produced a significant transient increase in fluorescence, which was greatly decreased by the NAADP-pathway antagonist Ned-19 and verapamil (Fig. 3a). We then used TET and its analogue CEP, both of which are bisbenzylisoquinoline alkaloids (BBAs) known for their potent anti-enterovirus activity [27]. Additionally, TET has been shown to inhibit NAADP-dependent Ca^2+^ signalling [19]. Our results demonstrated that both TET and CEP significantly reduced the NAADP-dependent Ca^2+^ signal, with CEP exhibiting a more pronounced inhibitory effect (Fig. 3a). Furthermore, the NAADP-AM–induced Ca^2+^ signal was significantly diminished in both TPC1-KO and TPC2-KO cells compared to parental HeLa cells (Fig. 3b). Overall, the data presented in Figs1^3^ indicate that the TPC-dependent, NAADP-evoked Ca^2+^ signal, which is influenced by pharmacological inhibition and genetic KO, is crucial for the replication of EV71.

### TPC1 or TPC2 deficiency does not affect EV71 binding or internalization

**Fig. 4.**
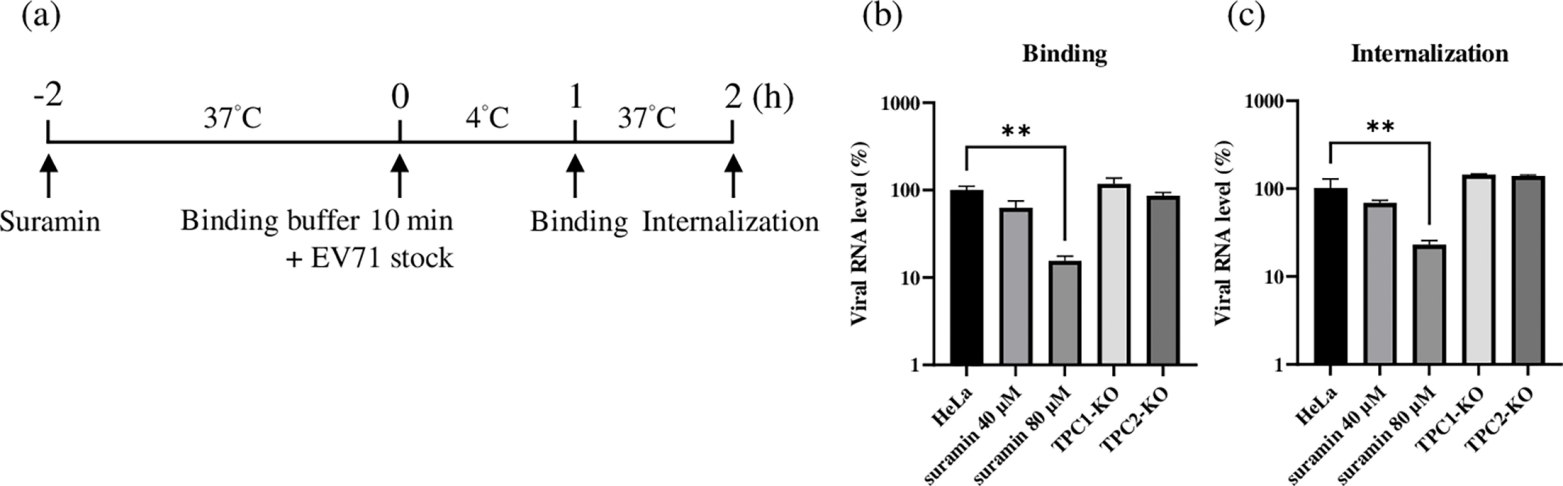
TPC1 or TPC2 deficiency does not affect EV71 binding or internalization. (**a**) Schematic timeline of the binding and internalization assays. HeLa cells were pretreated with suramin (40 or 80 µM) or DMSO for 2 h at 37 °C, transferred to 4 °C and incubated in binding buffer for 10 min. Next, 50 m.o.i. of EV71 was added to the cells (set as 0 h point), and the mixture was incubated at 4 °C for 1 h to allow the virus to adsorb onto the cells. For the binding assay, unbound virus was washed away with cold PBS, and the cells were treated with trypsin to remove any virus that was bound to the cell surface. For the internalization assay, following the initial adsorption of the virus at 4 °C, the cells were incubated at 37 °C for an additional hour to facilitate viral internalization. The cells were then washed with PBS and treated with trypsin again to eliminate any remaining virus on the cell surface. Finally, the viral RNA levels from both assays were quantified using RT-qPCR. RNA levels were normalized to human β-actin as an internal control. The results are presented as relative levels compared to a compound-free control containing 0.05% DMSO. The results for (**b**) the binding assay and (**c**) the internalization assay are shown. Data are shown as mean±sd from three independent experiments. Statistical significance was assessed by one-way ANOVA; **P<0.01.

We aimed to identify which steps of the EV71 life cycle are affected by the loss of either TPC gene. To achieve this, we first investigated whether virus binding and internalization were impacted in TPC1-KO or TPC2-KO cells. For the binding assay, we incubated the KO cells and their parental cells with EV71 at 4 °C for 1 h, allowing the virus to attach to the cells without permitting internalization. We then initiated internalization by shifting the temperature to 37 °C for an additional hour, which facilitates the uptake of the virus that was bound to the cell surface (Fig. 4a). Any virions that had not been internalized were removed using trypsin treatment. HeLa cells were also treated with suramin, a polysulfonated compound known to bind to the EV71 capsid and interfere with virus binding and internalization [34]. We measured viral RNA levels using RT-qPCR for each condition. Suramin reduced EV71 RNA levels in both the binding and internalization conditions in a dose-dependent manner. In contrast, the EV71 RNA levels recovered from TPC1-KO and TPC2-KO cells were comparable to those from parental HeLa cells (Fig. 4b, c). Together, these results indicate that loss of TPC1 or TPC2 does not affect viral binding or internalization.

### Loss of TPC1 or TPC2 impairs EV71 motility in the endolysosomal pathway

**Fig. 5.**
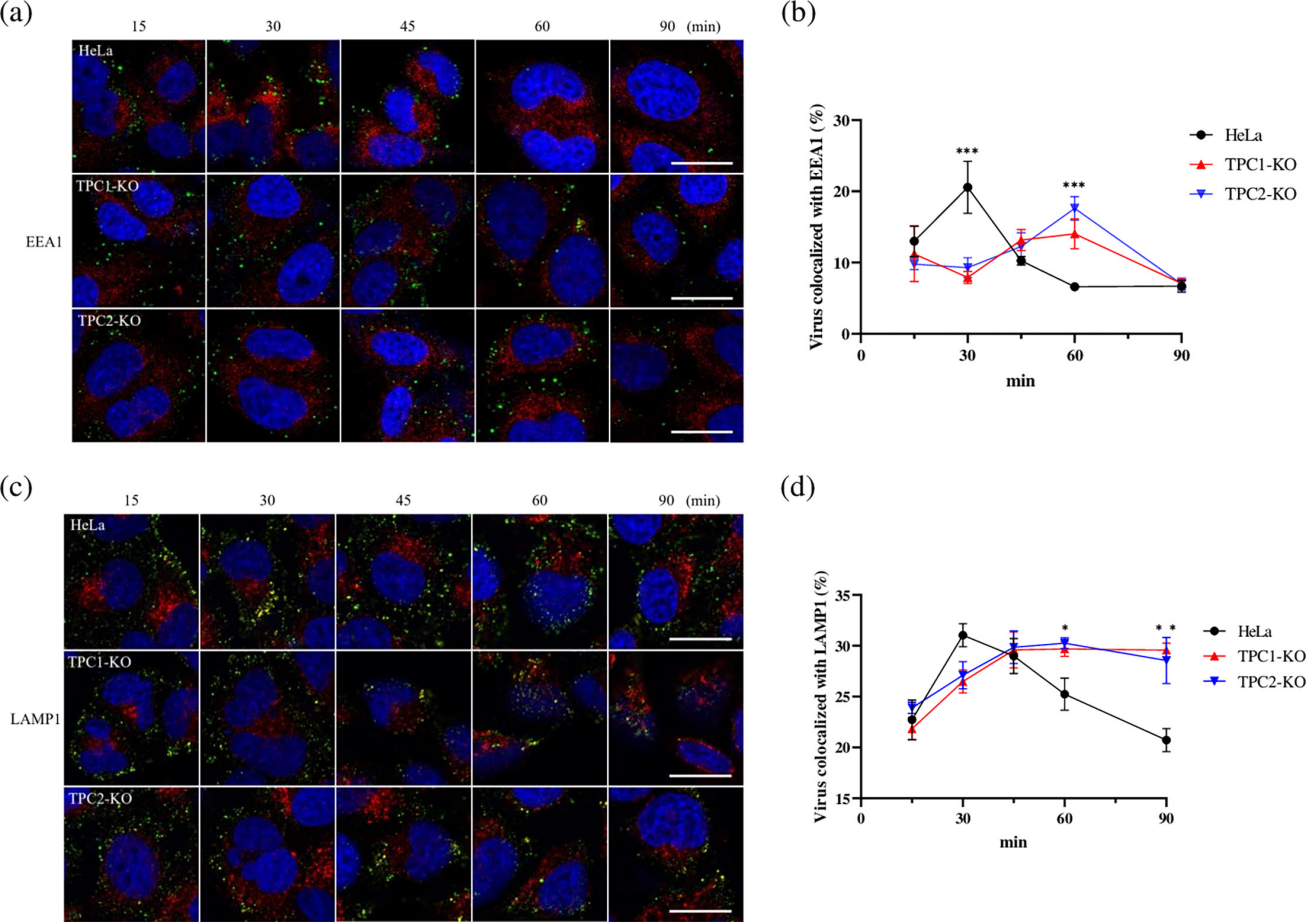
Loss of TPC1 or TPC2 delays EV71 progression through early and late endosomal compartments. (**a, b**) TPC1-KO and TPC2-KO cells, along with their parental HeLa cells, were inoculated with 300 m.o.i. of EV71 at 4 °C for 1 h to allow for synchronized binding. After this, the cells were shifted to 37 °C. At specified time points following the temperature shift, the cells were fixed and permeabilized. Viral antigen was detected using a rabbit anti-VP2 antibody, followed by an Alexa Fluor 488-conjugated secondary antibody (green). The nuclei were stained with DAPI (blue). Additionally, the cells were co-stained with an anti-EEA1 antibody (red) to label early endosomes. (**a**) Representative confocal images are presented for time points of 15, 30, 45, 60 and 90 min. (**b**) The quantification of the percentage of VP2 signal colocalized with EEA1 is shown (mean±sem). In parallel, the cells were also co-stained with an anti-LAMP1 antibody (red) to label late endosomes or lysosomes. (**c**) Representative confocal images for this staining at 15, 30, 45, 60 and 90 min are provided. (**d**) The quantification of the percentage of VP2 signal colocalized with LAMP1 is shown (mean±sem). For both quantifications, more than 200 cells were analysed per condition from at least three independent experiments. Statistical significance was evaluated by one-way ANOVA; ***P<0.001, **P<0.01, *P<0.05. Scale bars, 20 µm.

Given that viral binding and internalization are not reduced in TPC1-KO or TPC2-KO cells (Fig. 4), we next investigated whether either TPC protein is involved in a post-internalization trafficking step within the endolysosomal network. To examine this, we analysed the localization of the viral protein in relation to EEA1, an early endosome marker [35], and LAMP1, a late endosome and lysosomal marker [36]. Confocal microscopy revealed dynamic colocalization of EV71 VP2 with EEA1 in parental HeLa cells shortly after infection, followed by a gradual decline as the virions progressed to later compartments (Fig. 5a, b). In contrast, both TPC1-KO and TPC2-KO cells exhibited prolonged retention of EV71 in EEA1-positive early endosomes. Similarly, the colocalization of EV71 VP2 with LAMP1 peaked earlier in parental cells, but this was significantly delayed and reduced in both KO cell lines (Fig. 5c, d). These observations indicate that deletion of either TPC isoform leads to prolonged retention of viral particles within EEA1- and LAMP1-positive vesicles, suggesting that TPCs contribute to the efficient trafficking of EV71 through the endolysosomal system.

### TPC1 and TPC2 are not required for EV71 RNA translation and replication

**Fig. 6.**
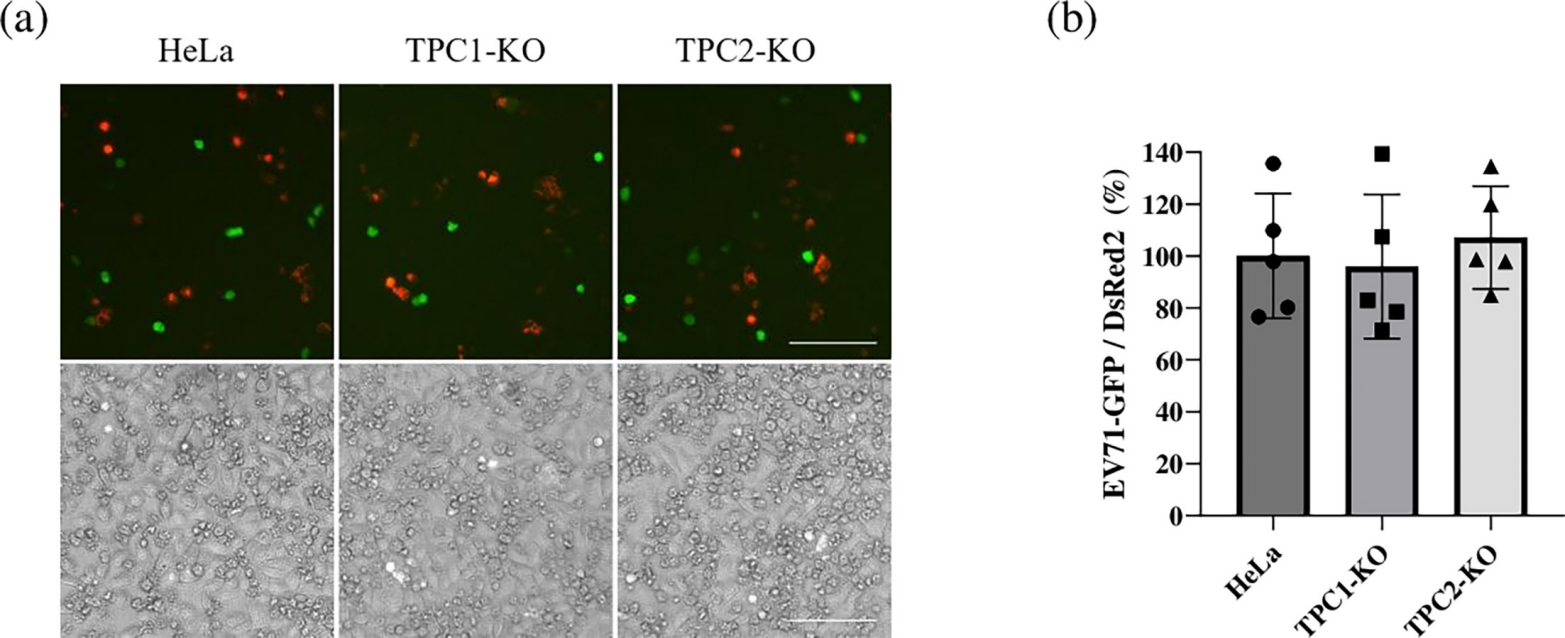
Depletion of TPC1 or TPC2 cells does not affect EV71 replicon-driven reporter expression. HeLa, TPC1-KO, and TPC2-KO cells were transfected with *in vitro*-transcribed EV71-GFP RNA generated from the pSVA-EV71-GFP plasmid, along with pDsRed2-N1 plasmid as a control for transfection efficiency over a period of 18 h. (**a**) Representative images of the reporter signal (green), the transfection control (red) and their phase contrast are shown. (**b**) The number of EV71-GFP-positive cells was normalized by the DsRed2-positive cells (EV71-GFP/DsRed2) and presented as relative levels compared to parental HeLa cells. Data are presented as mean±sd from independent experiments (*n*≥5). Statistical analysis was performed using one-way ANOVA; ns, not significant. Scale bar, 100 µm.

To investigate whether TPCs influence the replication of EV71 after the viral genome is delivered to the cytosol, we used an EV71 replicon that expresses GFP [28]. By directly transfecting *in vitro*-transcribed EV71-GFP RNA, we bypassed the processes of viral entry and uncoating. To monitor transfection efficiency, we cotransfected cells with pDsRed2-N1 plasmid which emits red fluorescence. Our findings showed that the ratio of GFP-positive cells to the transfection control cells was similar across parental, TPC1-KO and TPC2-KO cells (Fig. 6a, b). These results suggest that TPC1 and TPC2 do not influence viral RNA translation or replication once the viral genome is present in the cytoplasm. Furthermore, we performed a time-of-addition analysis using Ned-19, which revealed that inhibition was most effective during the entry-phase treatment but was minimal after the entry phase (Fig. S3). These findings are consistent with results from studies conducted with TPC1-KO and TPC2-KO cells (Figs4^6^).

### Ned-19 treatment and TPC1-KO or TPC2-KO suppress replication of multiple enterovirus serotypes

**Fig. 7.**
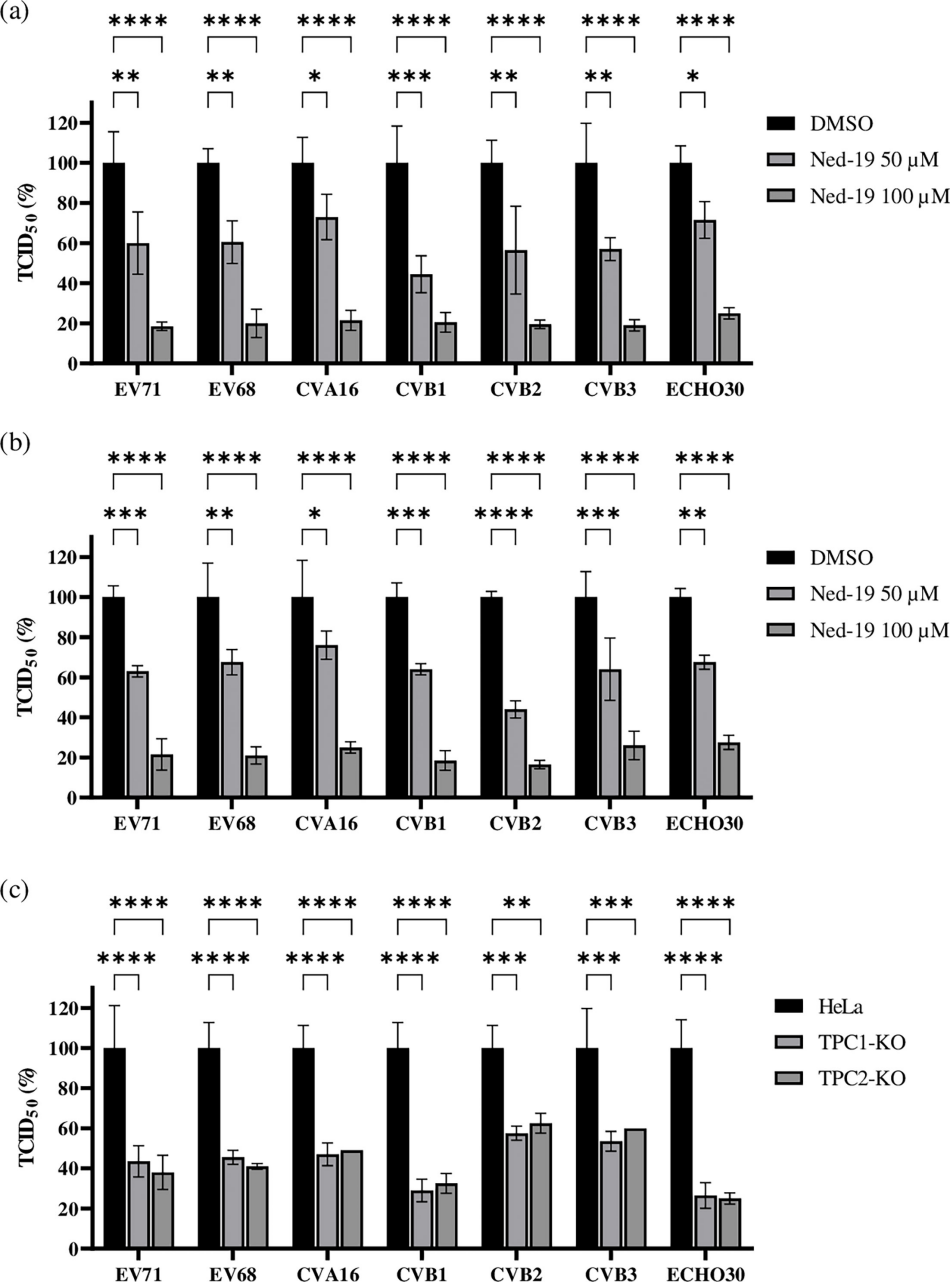
The treatment with Ned-19 and a deficiency in either TPC lead to a decrease in the replication of multiple enterovirus serotypes. (**a**) RD cells and (**b**) HeLa cells were pretreated with DMSO or Ned-19 (50 or 100 µM) for 1 h and then infected with the indicated enteroviruses, including EV71, EV68, CVA16, CVB1, CVB2, CVB3 and ECHO 30, at an m.o.i. of 0.1. (**c**) Parental HeLa, TPC1-KO and TPC2-KO cells were infected with the same virus panel (m.o.i.=0.1). For all infections, cell lysates and supernatants were collected at 8 h post-infection, and total virus titres were determined by the TCID_50_ assay. Data are presented as mean±sd (*n*=3). Statistical significance was evaluated by one-way ANOVA; ****P<0.0001, ***P<0.001, **P<0.01, *P<0.05.

To assess whether pharmacological interference with NAADP-associated signalling can broadly limit enterovirus replication, we investigated the effects of the NAADP antagonist Ned-19 in both RD and HeLa cells. The cells were pretreated with Ned-19 at concentrations of 50 or 100 µM, followed by infection with a variety of enteroviruses, including EV71, EV68, CVA16, CVB1, CVB2, CVB3 and Echo30. Viral titres were measured at 8 h post-infection, revealing a significant, dose-dependent reduction in infectious titres for all serotypes in RD cells (Fig. 7a) and a similar effect in HeLa cells (Fig. 7b). For most viruses, treatment with 50 µM Ned-19 decreased viral production to ~40–70% of the DMSO control, while treatment with 100 µM Ned-19 further reduced the titres to ~10–30%. These differences were statistically significant when compared to vehicle-treated cells.

We next investigated whether the genetic disruption of TPC channels produces an effect similar to the broad antiviral activity observed with Ned-19. Parental HeLa cells, along with TPC1-KO and TPC2-KO cells, were infected with various serotypes of enteroviruses, and their titres were measured after an 8 h infection period. The results indicated that the production of progeny viruses was consistently lower in both TPC1-KO and TPC2-KO cells compared to the parental HeLa cells (Fig. 7c). The degree of inhibition varied depending on the specific virus, but in general, the titres in the KO lines were reduced to ~30–60% of the levels observed in the parental cells. Overall, these findings support the idea that TPC-dependent endolysosomal functions represent a conserved host dependency factor for diverse enteroviruses.

### Transcriptomic profiling of TPC1-KO and TPC2-KO cells highlights endocytosis-related pathway enrichment

**Fig. 8.**
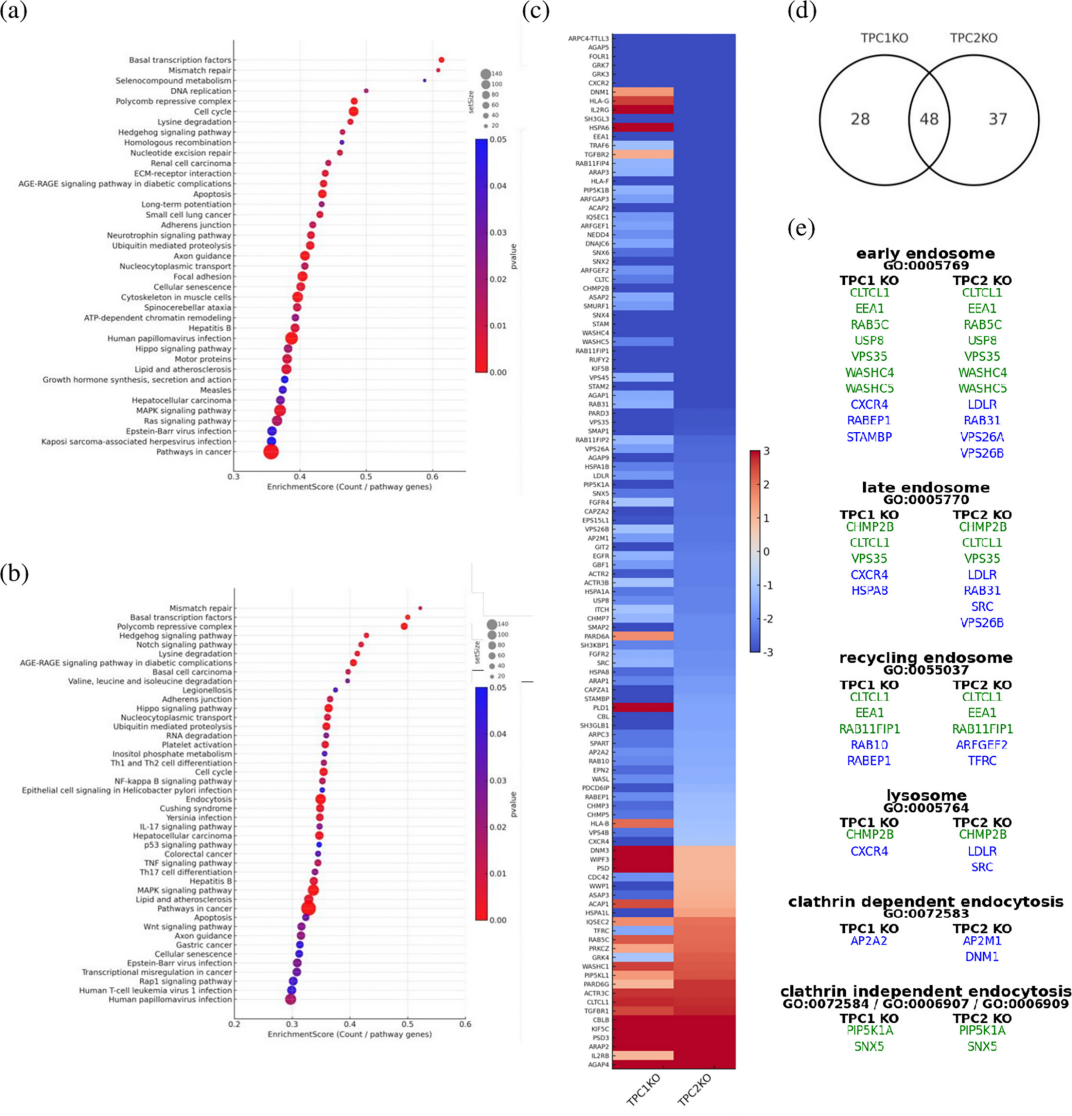
Transcriptomic profiling of TPC1-KO and TPC2-KO cells reveals enrichment of endocytosis-related pathways. KEGG pathway analysis of genes differentially expressed (a fold change of ≥2) in TPC1-KO (**a**) or TPC2-KO cells (**b**) relative to parental HeLa cells. Pathway analysis was performed using DAVID based on KEGG annotations. Each bubble represents one KEGG pathway; the x-axis indicates enrichment, and bubble size is proportional to the number of genes assigned to the pathway. Only pathways meeting the significance threshold (*P*<0.05) are shown. (**c**) Heatmap of expression changes for genes in the KEGG endocytosis pathway (map04144) in TPC1-KO and TPC2-KO cells relative to parental HeLa cells. Values are shown as log_2_ fold change (KO cells/parental cells); red and blue indicate increased and decreased expression, respectively. (**d**) Venn diagram showing overlap of endocytosis-pathway genes (map04144) in TPC1-KO and TPC2-KO cells relative to parental HeLa cells. (**e**) Summary of the gene categories associated with endocytosis and endolysosomes. The genes are organized by compartment: early endosome, late endosome, recycling endosome, lysosome, clathrin-dependent endocytosis and clathrin-independent endocytosis. Genes affected in both TPC1-KO and TPC2-KO cells (represented in green) were primarily linked to essential endolysosomal functions. In contrast, genes that were uniquely altered in either KO (shown in blue) were found across specific endocytic pathways.

To investigate the broader cellular changes associated with TPC loss, we conducted RNA-Seq in TPC1-KO and TPC2-KO cells, identifying differentially expressed genes with a fold change of ≥2 compared to their parental cells. Raw data from RNA-Seq is available in the online Supplementary Material (Table S1). KEGG pathway enrichment analysis revealed significant changes in pathways related to endocytosis in TPC2-KO but not in TPC1-KO lines (Fig. 8a, b). Notably, several highly ranked pathways associated with endocytic trafficking, including mitogen-activated protein kinase signalling, extracellular matrix-receptor interaction, focal adhesion and Ras signalling, were identified [37,41]. This prompted a focused examination of genes associated with endocytosis and endolysosomal processes.

The heatmap for the KEGG endocytosis pathway (map04144) demonstrated a significant decrease in the levels of endocytosis pathway transcripts, particularly pronounced in TPC2-KO cells (Fig. 8c). A Venn analysis of the genes associated with map04144 revealed a substantial core overlap of 48 genes that were altered in both TPC1-KO and TPC2-KO cells. Additionally, there were smaller subsets of genes that were preferentially altered: 28 genes in TPC1-KO cells and 37 genes in TPC2-KO cells (Fig. 8d). This indicates that while there is a significant overlapping response, there are also minor differences between the two isoforms. GO analysis revealed that many of the altered transcripts were linked to key trafficking and sorting modules, including early endosomes, late endosomes, lysosomes and both clathrin-dependent and -independent endocytic pathways (Fig. 8e). These transcriptomic changes are consistent with impaired endolysosomal trafficking, providing a mechanistic framework that connects TPC function to the entry of enteroviral infections.

## Discussion

In this study, we identify endolysosomal TPCs, TPC1 and TPC2, as critical host factors that promote enterovirus infection by facilitating viral trafficking through the endocytic pathway. Using complementary pharmacological and genetic approaches, we demonstrate that inhibition or loss of either TPC isoform markedly suppresses EV71 replication (Figs1  2) and broadly restricts multiple enterovirus serotypes (Fig. 7). Mechanistically, TPCs are dispensable for viral binding, internalization (Fig. 4) and post-entry RNA replication (Fig. 6), but are required for efficient progression of incoming virions through early and late endosomal compartments (Fig. 5). These findings uncover a previously unappreciated role for TPC-dependent processes in the life cycle of non-enveloped enteroviruses.

Endolysosomal Ca^2+^ release has emerged as a key regulator of membrane fusion, fission and organelle maturation. TPCs, activated by NAADP, generate localized Ca^2+^ microdomains at endosomal and lysosomal membranes that coordinate trafficking events [13]. Our Ca^2+^ imaging data confirm that both TPC1 and TPC2 contribute to NAADP-evoked Ca^2+^ release in HeLa cells and that this signalling is effectively suppressed by Ned-19 and verapamil (Fig. 3). Importantly, disruption of this pathway phenocopies the antiviral effects observed upon genetic ablation of either TPC, strongly linking TPC-dependent Ca^2+^ signalling to enterovirus infection (Fig. 3). It is noted that TPCs can also be activated by the PI(3,5)P₂ to release sodium ions, which helps regulate the membrane potential, vesicular trafficking and endolysosomal homeostasis. However, the activation is not specific as other lysosomal cation channels such as transient receptor potential mucolipin channels (TRPMLs) are also activated by PI(3,5)P₂ [42,43]. In our previous study, berbamine, a BBA reported to inhibit TRPML channels, exhibited significantly weaker antiviral activity against EV71 when compared to CEP and TET [27]. While TRPML channels play a role in receptor recycling for SARS-CoV-2 and the Japanese encephalitis virus [44], these findings suggest that EV71 infection depends more on TPC pathways than on TRPML pathways.

The endosomal itinerary of EV71 requires progressive maturation from early to late endosomes, where acidification and receptor-mediated conformational changes promote capsid uncoating. Our confocal microscopy analyses reveal that in the absence of TPC1 or TPC2, EV71 particles are retained in early endosomes and exhibit delayed or reduced access to LAMP1-positive late endosomal and lysosomal compartments (Fig. 5). These observations support a model in which TPC-mediated Ca²⁺ release facilitates endosomal maturation or fusion events necessary for efficient viral transport to uncoating-competent compartments. In addition to local Ca²⁺ signalling, TPC-dependent endolysosomal function may also intersect with cholesterol homeostasis. Previous studies have demonstrated that a deficiency in TPC2 impairs the trafficking processes along the endolysosomal degradative pathway. This includes the defective transport of low-density lipoprotein-cholesterol and epidermal growth factor (EGF)/EGF-receptor cargo, and this impairment is associated with a reduced ability for late endosomes to fuse with lysosomes [45,46]. Additionally, the activation of TPC2 using the specific agonist has been shown to improve cholesterol accumulation phenotypes in models of lysosomal storage disease that have impaired TPC2 function [47]. Furthermore, the homeostasis of membrane cholesterol is closely linked to endocytosis and intracellular membrane trafficking [48]. It has also been established that infections by several enveloped and non-enveloped viruses rely on an intact membrane cholesterol environment [49,51]. Consequently, we hypothesize that TPC deficiency or inhibition could disrupt endolysosomal cholesterol distribution, as demonstrated by Filipin staining that colocalizes with LAMP1-positive regions, subsequently affecting the entry and trafficking of various viruses, including EV71 [51,52]. However, this connection remains a mechanistic inference supported by literature and will require further experimental validation.

Although EV71 served as the primary model in this study, our findings extend to a broad panel of enteroviruses, including EV68, coxsackieviruses A and B, and echovirus 30. Both pharmacological inhibition and genetic ablation of TPCs significantly reduced replication of all tested serotypes, indicating that reliance on TPC-mediated endolysosomal trafficking is conserved across the genus. This broad requirement likely reflects the shared need among enteroviruses for orderly endosomal maturation to trigger uncoating, despite differences in receptor usage and tissue tropism.

Previous studies have established essential roles for TPCs in the entry of enveloped viruses such as Ebola virus and coronaviruses, where TPC inhibition blocks viral trafficking and membrane fusion within endolysosomes. However, the processes involved in viral binding and internalization during the entry stage, as well as subsequent post-entry stages of the infectious cycle, have not been explored [19,20]. In contrast, a key strength of our work is the precise mapping of TPC function to a discrete stage of the viral life cycle. Moreover, this study broadens the paradigm to include non-enveloped viruses. It demonstrates that TPCs are essential not only for the fusion of virus and cell membranes specific to enveloped viruses but also for trafficking steps that precede capsid uncoating during the entry of non-enveloped viruses. This finding expands the conceptual understanding of TPC function in viral infections and emphasizes endolysosomal Ca^2+^ signalling as a common regulatory mechanism utilized by various viral families.

Some findings in this study are particularly noteworthy, given the lack of approved antivirals for enterovirus infections and the limited cross-protective capacity of existing vaccines. TET has been shown to inhibit NAADP-evoked Ca²⁺ release [19]. In this study, we report for the first time that CEP, an analogue of TET, demonstrates an even stronger inhibitory effect on NAADP-evoked Ca²⁺ release, which aligns with its more potent antiviral activity against enteroviruses in cell cultures [27]. BBAs consist of two benzylisoquinoline units connected by one to three diaryl ether bridges. Structure-activity relationship (SAR) analysis suggests that subtle structural variations, particularly ether-bridge connectivity, may significantly influence antiviral potency, potentially through TPC block. Specifically, BBAs with a 7-to-8′ phenolic *O*-linkage, such as CEP, show significantly higher antiviral activity compared to compounds that have an 8-to-7′ *O*-linkage, like TET, fangchinoline and berbamine (Fig. S4) [27,53]. These findings indicate that ether-bridge connectivity is an important structural determinant of antiviral efficacy, although the precise SAR underlying antiviral activity and TPC block requires further investigation. CEP is an approved drug in Japan for treating certain acute and chronic diseases, including leucopenia, snake bites and alopecia [54,55]. Furthermore, CEP has been shown to effectively inhibit enterovirus infections in a mouse model of EV71 infection [27]. Understanding that the NAADP-dependent Ca²⁺ signalling contributes to CEP’s antiviral mechanisms is essential for repositioning it as an antiviral treatment against enterovirus infections. Additionally, EV71 replicon assays have demonstrated that viral RNA translation and replication proceed normally in TPC-deficient cells once the genome is delivered to the cytosol (Fig. 6). This finding highlights that TPCs operate upstream of genome release. This distinction between entry trafficking and downstream replication is conceptually important, as it sets TPCs apart from traditional antiviral targets that inhibit viral enzymes or genome synthesis. Instead, TPCs act as host regulators of Ca²⁺ signalling, offering a promising strategy for developing broad-spectrum anti-enteroviral therapies that may reduce the likelihood of viral resistance.

For the first time, we report a side-by-side comparison of the global transcriptome analysis of cells depleted of TPC1 and TPC2. Disruption of either channel leads to coordinated changes in the expression of genes related to endocytosis (Fig. 8). This supports the idea that TPC-mediated Ca²⁺ signalling plays a role in maintaining homeostasis in endosomal trafficking. Importantly, the incomplete overlap in transcriptional responses between TPC1-KO and TPC2-KO cells suggests that the two channels are not fully redundant. This functional divergence aligns with their known subcellular distributions: TPC1 is primarily associated with early endosomal compartments, while TPC2 is enriched in late endosomes and lysosomes [15,16]. Thus, disturbances in different stages of endocytosis may trigger adaptive transcriptional programmes specific to each compartment. Moreover, endocytic trafficking and endosomal maturation are essential for the entry and intracellular trafficking of many viruses [56,57]. Thus, the transcriptional reprogramming observed upon loss of TPC1 or TPC2 provides a mechanistic framework for understanding how TPCs influence viral infection. Future studies using primary human cells or *in vivo* models will validate the physiological relevance of TPC-dependent enterovirus restriction.

In summary, our study identifies TPC1 and TPC2 as crucial host factors that influence enterovirus infection by regulating endolysosomal trafficking during the viral entry process. By acting at a conserved host-encoded step that occurs before viral genome release, TPCs are promising targets for developing broad-spectrum antiviral treatments against enteroviruses. Additionally, our discovery of CEP as an inhibitor of NAADP-dependent Ca^2+^ signalling enhances its potential for translation into therapeutic use. Our transcriptome analysis supports a model in which TPC1 and TPC2 work together to shape the endocytic and endolysosomal environment while exerting isoform-specific control over different trafficking pathways. This regulation is crucial for cellular processes that facilitate viral entry. More broadly, our findings expand the significance of TPC-regulated endolysosomal signalling from the context of enveloped viruses to non-enveloped enteroviruses, emphasizing that TPC-dependent Ca^2+^ dynamics serve as a central link between intracellular trafficking and infection outcomes.

## Supplementary material

10.1099/jgv.0.002294 Supplementary Material 1.

10.1099/jgv.0.002294 Table S1.

## Abbreviations

BBA: bisbenzylisoquinoline alkaloid
CEP: cepharanthine
CVA16: coxsackievirus A16
DAVID: Database for Annotation, Visualization and Integrated Discovery
Echo30: Echovirus serotype 30
EEA1: early endosome antigen 1
EGF: epidermal growth factor
EV71: enterovirus 71
GO: gene ontology
IFA: immunofluorescence assay
KEGG: Kyoto Encyclopedia of Genes and Genomes
KO: knockout
LAMP1: lysosome-associated membrane protein 1
MEM: minimum essential medium
NAADP: nicotinic acid adenine dinucleotide phosphate
RD: Rhabdomyosarcoma
RNA-Seq: RNA sequencing
RT-qPCR: reverse transcription quantitative polymerase chain reaction
SAR: structure-activity relationship
TET: tetrandrine
TPC: two-pore channel
