# Supplementary material for: Endolysosomal two-pore channels regulate a conserved entry pathway for enteroviruses
Source: J Gen Virol. 2026 Jul 15;107(7):002294. doi: 10.1099/jgv.0.002294 (PMC13372183; doi:10.1099/jgv.0.002294)
Supplement: Supplementary Material 1. [file jgv-107-02294-s001.pdf]

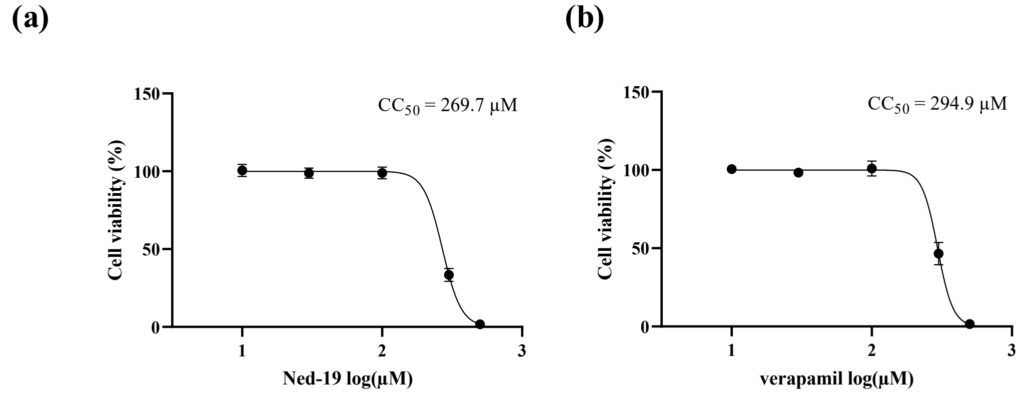

FIG. S1. Cytotoxicity of Ned-19 and verapamil in HeLa cells. HeLa cells were treated with Ned-19 (a) or verapamil (b) for 12 h. Cell viability was measured using the CellTiter 96 AQueous Cell Proliferation Assay (Promega) and expressed relative to vehicle-treated cells (0.05% DMSO). CC_{50} values were determined by nonlinear regression using GraphPad Prism 9 and are indicated on the plots. Data are presented as the mean ± SEM from three independent experiments.

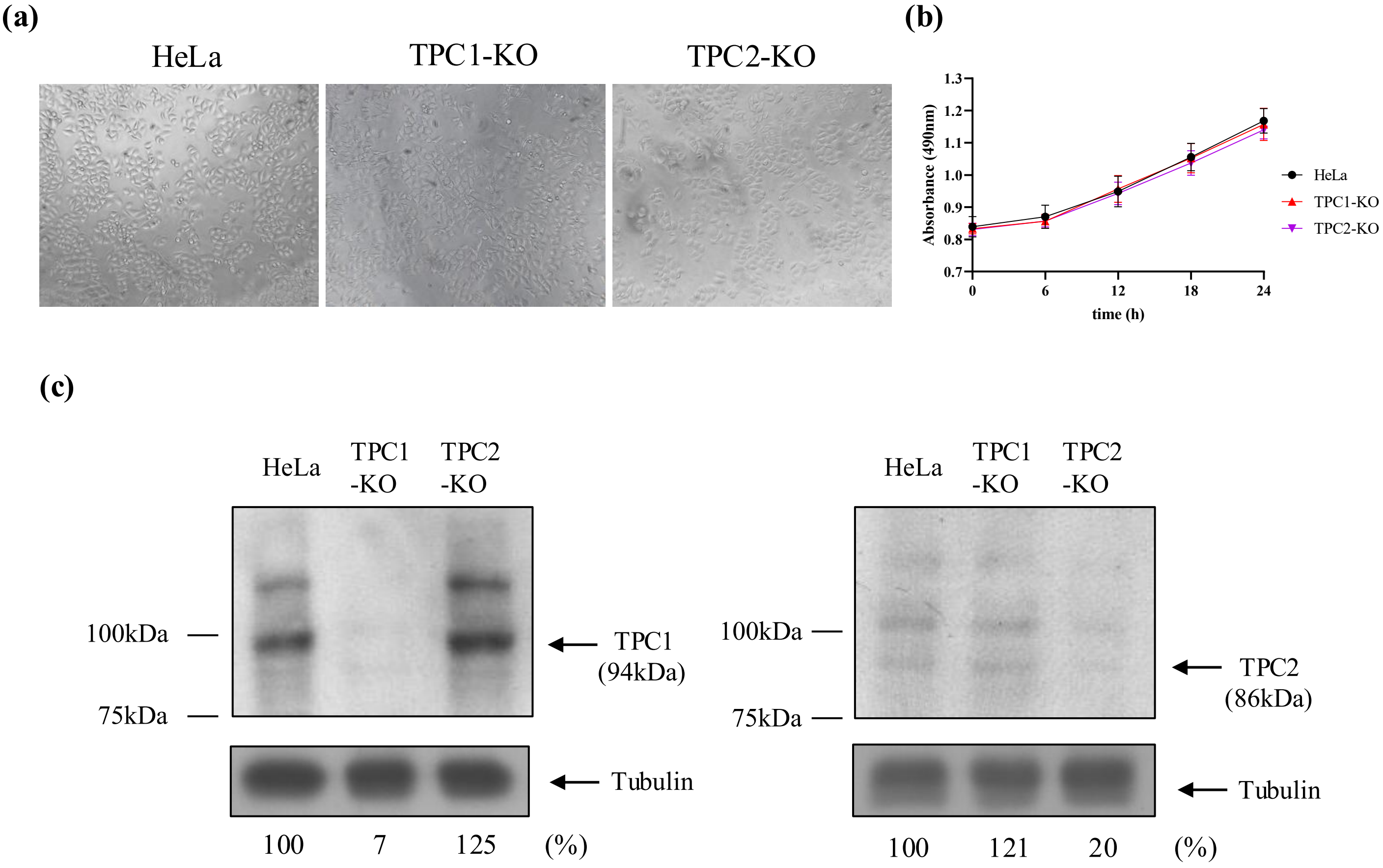

Figure S2. Parental HeLa, TPC1-KO, and TPC2-KO cells display comparable morphology, growth kinetics, along with selective abrogation of TPCs. (a) Representative phase-contrast images of parental HeLa, TPC1-KO, and TPC2-KO lines. (b) Cell growth was assessed by an MTS assay, and absorbance at 490 nm was measured at the indicated time points (0–24 h) for parental HeLa, TPC1-KO, and TPC2-KO cells. Data are presented as mean ± SD (n=3). (c) Western blot analysis was used to detect the endogenous TPC1 and TPC2 proteins in parental HeLa cells as well as in TPC1-KO and TPC2-KO cells, with tubulin serving as a loading control. The numbers below the tubulin bands represent the densitometric quantification of TPC1 and TPC2, normalized to tubulin and expressed as a percentage relative to the parental HeLa cells. The TPC1 and TPC2 proteins are sized at 94 kDa and 86 kDa, respectively. The bands above 100 kDa represent their various glycosylated forms (PMID: 21173144).

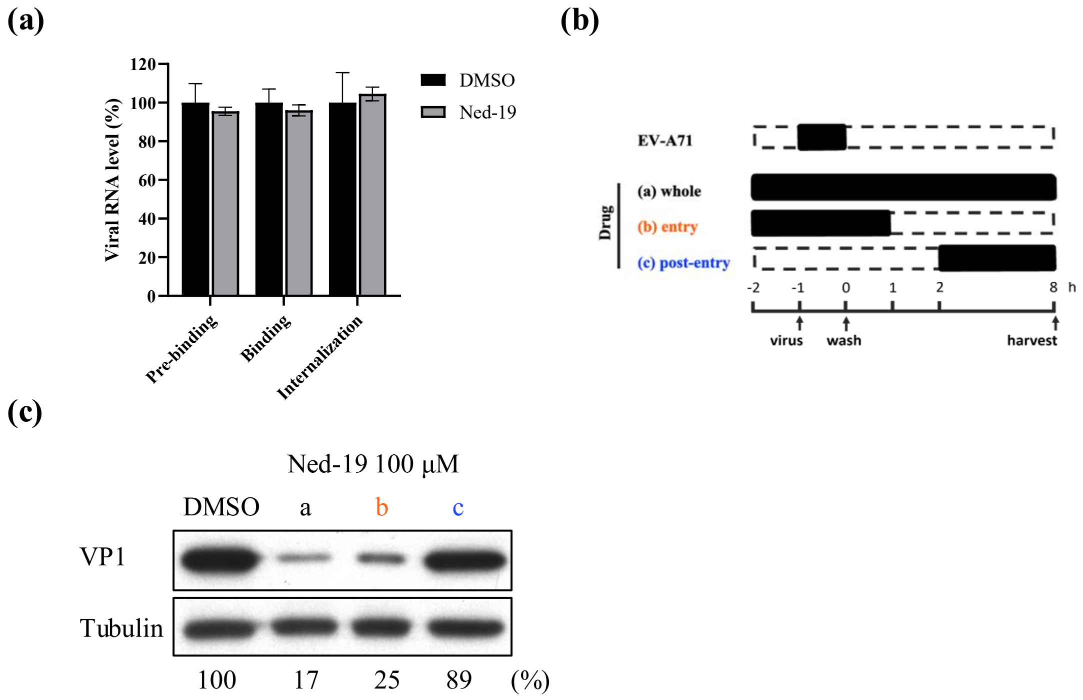

Figure S3. Timing of EV71 inhibition by Ned-19. (a) Effects of Ned-19 on EV71 pre-binding (virucidal), binding, and internalization. To evaluate the direct virucidal activity against EV71, virus stocks (1000 TCID$_{50}$) were incubated with 1 mM Ned-19 in serum-free medium for 1 hour at 37°C. After incubation, the virus-compound mixtures were diluted 100-fold to minimize any residual effects of the compound before proceeding with the infection. The treated virus was then inoculated onto RD cells and allowed to adsorb for 1 hour at 37°C. For the binding assay, HeLa cells were pre-treated with 100 μM of Ned-19 in a 37°C incubator for 2 hours. Following this, the cells were placed on ice and treated with 1 mL of binding buffer (PBS containing 1% BSA and 0.1% sodium azide) for 10 minutes. Then, 50 MOI of EV-71 was added, and the mixture was incubated on ice for 1 hour to allow the virus to adsorb to the cells. After this, unbound virus was washed off with PBS at 4°C, and the cells were treated with trypsin to detach any virus that had bound to the cell surface. For the internalization assay, after the initial adsorption of the virus on ice, the cells were incubated at 37°C for an additional hour to enable viral internalization. Subsequently, the cells were washed with PBS and treated with trypsin to remove any remaining virus on the cell surface. After completing the viral adsorption for 8 hours, we quantified viral RNA levels from all three assays using RT-qPCR, and normalized to the DMSO controls. Data are presented as mean ± SD from three independent experiments. (b) Schematic diagram of the time-of-addition assay. HeLa cells were infected with EV71, and Ned-19 (100 μM) was added either throughout the entire experiment (whole), during the entry stage, or during the post-entry stage, as indicated. Virus adsorption, wash, and harvest time points are shown below the timeline. (c) Representative immunoblot analysis of EV71 VP1 expression under the indicated treatment conditions in the time-of-addition assay, with tubulin as the loading control. DMSO served as the control. The percentages shown below the bands represent densitometric quantification relative to the DMSO control lane (set to 100%).

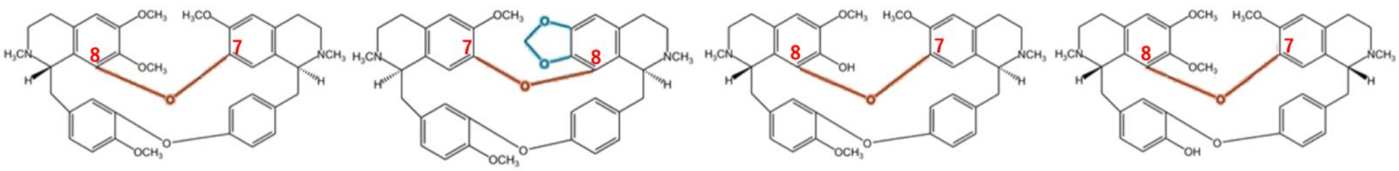

Tetrandrine (TET) Cepharanthine (CEP) Fangchinoline (FAN) Berbamine (BER)

Fig. S4 A structural comparison of BBAs and their phenolic O-linkage connectivity is presented. The chemical structures of TET, CEP, FAN, and BER are depicted. The orange lines highlight the phenolic O-linkages that connect the two benzylisoquinoline units, while the red numbers indicate the positions of these linkages. CEP features a 7-to-8′ phenolic O-linkage, whereas TET, FAN, and BER possess an 8-to-7′ O-linkage. Additionally, the blue highlight marks the 6′,7′-dioxolane moiety of CEP.
